# Renal Cell Carcinoma After the COVID-19 Pandemic: Evidence of More Advanced Pathological Presentation from a Romanian Tertiary Center

**DOI:** 10.3390/cancers18172812

**Published:** 2026-08-30

**Authors:** Adelina Vidac, Adrian Văduva, Robert Barna, Bianca Natarâș, Aura Jurescu, Ioana Hurmuz, Diana Nicolcea, Vlad Dema, Silviu Lațcu, Alin Cumpănaș, Alis Dema

**Affiliations:** 1 ANAPATMOL Research Center, “Victor Babes” University of Medicine and Pharmacy, 300041 Timisoara, Romania; robert.barna@umft.ro (R.B.); dema.alis@umft.ro (A.D.); 2 Department of Pathology, “Victor Babes” Clinical Hospital of Infectious Diseases and Pulmonology, 300310 Timisoara, Romania; 3 Department of Pathology, “Pius Brinzeu” Emergency County Hospital, 300723 Timisoara, Romania; 4 Department of Urology, “Pius Brinzeu” Emergency County Hospital, 300723 Timisoara, Romania; 5 Department of Urology, “Victor Babes” University of Medicine and Pharmacy, 300041 Timisoara, Romania

**Keywords:** renal cell carcinoma, COVID-19 pandemic, pathology, retrospective study

## Abstract

**Background/Objectives:** The COVID-19 pandemic disrupted healthcare systems worldwide, raising concerns about delayed cancer diagnosis and treatment. This study evaluated whether renal cell carcinoma (RCC) diagnosed after the pandemic showed more advanced pathological features at presentation. **Methods:** This retrospective study included all partial and radical nephrectomy specimens with a histopathological diagnosis of RCC examined between March 2018 and February 2023, stratified into pre-COVID (2018–2020), COVID (2020–2021), and post-COVID (2021–2023) periods. Pathological variables were compared using Pearson’s χ^2^ and Kruskal–Wallis tests; odds ratios with 95% confidence intervals were calculated with Holm-Bonferroni correction, and multivariable logistic regression, adjusting for age, sex, tumor size, residence, and histological subtype, was performed for high pathological stage and vascular invasion. **Results:** A total of 297 RCC cases were included (121 pre-COVID, 46 COVID, 130 post-COVID). Post-COVID tumors more frequently showed advanced pathological stage (61.5% vs. 41.3% and 34.8%; *p* < 0.001) and vascular invasion (56.9% vs. 29.8% and 34.8%; *p* < 0.001) than pre-COVID and COVID tumors. These associations survived Holm-Bonferroni correction, except for the post-COVID vs. COVID vascular invasion comparison. Both associations remained significant, and numerically stronger, after multivariable adjustment (adjusted OR 2.91, 95% CI 1.61–5.25 for stage; adjusted OR 3.80, 95% CI 2.11–6.83 for vascular invasion). WHO/ISUP grade, tumor necrosis, and sarcomatoid/rhabdoid differentiation did not differ significantly among periods. **Conclusions:** This study demonstrates a temporal association between the post-pandemic period and more advanced RCC at surgery, independent of age, sex, tumor size, residence, and histological subtype. These findings are consistent with, though do not establish, pandemic-related delays in diagnosis and treatment as a contributing factor, underscoring the need for studies incorporating institutional healthcare-utilization and outcome data.

## Simple Summary

The COVID-19 pandemic caused major disruptions to healthcare systems worldwide, delaying access to medical consultations, diagnostic imaging, and surgical treatment for many patients with cancer. Because renal cell carcinoma (RCC) is often detected incidentally during abdominal imaging, these disruptions may have affected the pathological presentation of tumors at the time of surgical treatment. In this retrospective study, we compared the pathological characteristics of RCC diagnosed before, during, and after the COVID-19 pandemic in a Romanian tertiary referral center. We found that tumors treated after the pandemic more frequently presented with advanced pathological stage and vascular invasion, both of which are associated with a poorer prognosis, while other markers of tumor aggressiveness, such as grade and necrosis, showed no significant change. These findings suggest that healthcare disruptions during the COVID-19 pandemic may have contributed to some patients presenting with more advanced RCC, though this study cannot prove a direct cause-and-effect relationship. The results underscore the need to preserve uninterrupted access to cancer diagnosis and treatment during future healthcare crises.

## 1. Introduction

The coronavirus disease 2019 (COVID-19) pandemic profoundly disrupted healthcare systems worldwide, resulting in substantial changes to healthcare delivery. In response to the rapid spread of severe acute respiratory syndrome coronavirus 2 (SARS-CoV-2), healthcare resources were redirected toward the management of infected patients, while elective procedures, outpatient consultations, and diagnostic investigations were frequently postponed or restricted [1,2,3]. Simultaneously, concerns regarding exposure to infection led many patients to delay seeking medical attention, further contributing to reduced access to healthcare services [2,3].

These disruptions raised concerns regarding their potential impacts on cancer diagnosis and treatment. Several studies have reported reductions in cancer screening, delays in diagnostic workups, postponement of surgical procedures, and interruptions in oncological care during the pandemic period [1,2,3]. Consequently, increasing attention has been directed toward determining whether these delays resulted in more advanced disease at presentation and potentially worse oncological outcomes [1,2,3].

Renal cell carcinoma (RCC) is the most common malignant neoplasm of the kidney and accounts for approximately 2–3% of all adult malignancies [4]. The widespread use of cross-sectional imaging has contributed to an increasing number of incidentally detected renal tumors, facilitating diagnosis at earlier stages [4,5]. However, many RCCs remain clinically silent until advanced disease develops, making timely access to imaging studies and urological evaluation essential for early detection and treatment [4,5]. Therefore, disruptions to healthcare services during the COVID-19 pandemic may have influenced both the stage and pathological characteristics of RCC at diagnosis.

Several studies have investigated the impact of the pandemic on RCC diagnosis and management, with conflicting results. Janes et al. reported a reduction in RCC diagnoses accompanied by an increase in patients presenting with pT3 tumors following the pandemic, suggesting a shift toward more advanced disease at presentation [6]. Similarly, Gupta et al. observed higher pathological stage, higher nuclear grade, and an increased prevalence of metastatic disease among patients diagnosed during the COVID era [7]. In contrast, Mangone et al. and Yildirim et al. reported a decrease in newly diagnosed RCC cases without a corresponding increase in advanced-stage disease, suggesting that the oncological consequences of the pandemic may vary according to healthcare infrastructure and regional factors [8,9].

Furthermore, most available studies have focused on surgical activity and treatment delays, while relatively few have specifically evaluated morphological markers of tumor aggressiveness. To our knowledge, data regarding the impact of the COVID-19 pandemic on the pathological presentation of RCC remain limited, particularly in Eastern European populations.

Given these conflicting findings, additional pathology-based studies are needed to clarify the long-term impact of pandemic-related healthcare disruptions on RCC presentation. Therefore, this study sought to determine whether the COVID-19 pandemic was associated with changes in the pathological presentation of RCC in a Romanian tertiary referral center. We found that patients treated after the pandemic more frequently presented with advanced pathological stage and vascular invasion. These findings are temporally associated with the post-pandemic period and are consistent with, though do not establish, delayed diagnosis as a contributing factor to more advanced disease at the time of surgery.

## 2. Materials and Methods

This retrospective, single-center study was conducted in the Department of Pathology of the “Pius Brinzeu” Emergency County Hospital Timisoara (SCJUPBT), Romania. The study included all partial and radical nephrectomy specimens with a histopathological diagnosis of RCC examined between 1 March 2018 and 28 February 2023. For the purpose of analysis, cases were stratified into three groups according to the timeline of the COVID-19 pandemic: pre-COVID (1 March 2018–28 February 2020), COVID (1 March 2020–28 February 2021), and post-COVID (1 March 2021–28 February 2023). Cases were additionally examined on a year-by-year basis across five annual intervals spanning the same period, to evaluate whether observed differences reflected a discrete pandemic-associated shift rather than a pre-existing secular trend. The study periods were defined according to the chronology of the COVID-19 pandemic and related public health restrictions in Romania, which included a national state of emergency in spring 2020 during which elective medical procedures were suspended [10], followed by recurring epidemic waves that continued to strain healthcare capacity through 2021 [11]. The COVID period represented the time during which healthcare services experienced the greatest disruption, whereas the post-COVID period reflected the gradual resumption of routine care.

Cases were identified through a search of the institutional pathology database using keywords such as “renal cell carcinoma”, “kidney cancer”, and “kidney tumor”. Inclusion criteria comprised all partial and radical nephrectomy specimens with a histopathological diagnosis of RCC. Cases diagnosed exclusively on biopsy, as well as other renal malignancies (e.g., urothelial carcinoma) and benign or borderline renal tumors (e.g., angiomyolipoma and oncocytic neoplasms), were excluded. No patients presented with bilateral RCC, and for patients with multiple ipsilateral tumors, the lesion with the highest pathological stage was selected for analysis.

A total of 297 cases met the inclusion criteria and were included in the study, comprising 121 cases in the pre-COVID group, 46 cases in the COVID group, and 130 cases in the post-COVID group.

Demographic and pathological data were collected from pathology reports and included patient age, sex, place of residence (urban vs. rural), tumor size, histological subtype, WHO/ISUP grade, tumor necrosis, sarcomatoid and/or rhabdoid differentiation, vascular invasion, and pathological tumor stage (pT). Histopathological diagnoses were established according to the 2016 and 2022 editions of the WHO Classification of Urinary and Male Genital Tumours. The 2022 edition primarily revised histological subtyping and nomenclature, introducing new molecularly-defined RCC entities and removing the type 1/2 subclassification of papillary RCC. Nuclear grading was assigned using the WHO/ISUP grading system, introduced in the 2016 WHO edition and unchanged in the 2022 revision. Pathological tumor stage (pT) was assigned according to the American Joint Committee on Cancer (AJCC)/Union for International Cancer Control (UICC) TNM staging system, 8th edition, which remained in effect without revision throughout the study period. Vascular invasion was further subclassified, where specified in the original pathology report, into renal sinus vascular invasion, segmental/renal vein invasion, microscopic vascular invasion, and unspecified vascular invasion. These categories were not mutually exclusive, as individual tumors could exhibit more than one type. For comparative analyses, tumors were categorized as low-grade (WHO/ISUP grades 1–2) or high-grade (WHO/ISUP grades 3–4), and as low-stage (pT1–pT2) or high-stage (pT3–pT4).

WHO/ISUP grade could not be assigned for 13 patients (5 pre-COVID, 1 COVID, 7 post-COVID): 10 had chromophobe RCC, a histological subtype not eligible for WHO/ISUP grading, and in the remaining 3 cases grade was not specified in the original pathology report. These 13 patients were excluded from grade-specific analyses.

All specimens had been processed as part of routine diagnostic practice using formalin fixation and paraffin embedding, with hematoxylin and eosin (H&E) staining. Histopathological data were extracted from original diagnostic reports, and no additional slide review was performed for the purpose of this study.

Statistical analyses were conducted using Jamovi (version 2.6; The Jamovi Project, Sydney, Australia). Categorical data were compared across groups using Pearson’s chi-square test, with results expressed as counts and percentages; continuous variables, which did not follow a normal distribution, were compared using the Kruskal-Wallis test and summarized as medians with interquartile ranges (IQRs). A two-sided *p*-value below 0.05 was considered indicative of statistical significance. Given that five pathological variables were compared across two pairwise contrasts (post-COVID versus pre-COVID, and post-COVID versus COVID), odds ratios (OR) and their corresponding 95% confidence intervals (CI) were derived using the normal approximation to the log-odds ratio for variables reaching significance on chi-square testing; the resulting *p*-values were then adjusted for multiple testing using the Holm-Bonferroni procedure, implemented via the *p*.adjust function in R (version 4.5.0; R Foundation for Statistical Computing, Vienna, Austria), accessed through the Rj Editor module within Jamovi. To further evaluate whether the association between study period and these outcomes was independent of other variables, multivariable logistic regression models were additionally constructed for high pathological stage and vascular invasion, with study period as the principal predictor of interest, adjusted for age, sex, tumor size, residence, and histological subtype (dichotomized as clear-cell versus non-clear-cell RCC given the small size of individual non-clear-cell categories). Adjusted odds ratios with 95% confidence intervals are reported, together with overall model fit statistics (likelihood-ratio χ^2^, Akaike Information Criterion, and McFadden’s R^2^). Sensitivity analyses using alternative definitions of the post-COVID period, restricted to individual study years, were additionally performed and are reported in the Section 3 (Supplementary Table S1).

Graphical illustrations were generated with the assistance of ChatGPT (GPT-5.5; OpenAI, San Francisco, CA, USA), based on data supplied by the study authors, and were subsequently checked for accuracy and finalized by the authors.

The study was conducted in accordance with the Declaration of Helsinki and received approval from the Ethics Committee of the Doctoral School of the “Victor Babes” University of Medicine and Pharmacy, Timisoara, Romania (Approval No. 94/1 October 2024). Given the retrospective design of the study and the use of anonymized data, the requirement for individual informed consent was waived. However, upon hospital admission, all patients provided general consent for the use of their medical data for research purposes.

## 3. Results

A total of 297 patients with histologically confirmed RCC were included in the study, comprising 121 pre-COVID cases, 46 COVID cases, and 130 post-COVID cases. Because the three periods were not of equal length, this corresponded to an average caseload of approximately 60.5 cases/year before the pandemic, 46 cases/year during the pandemic, and 65 cases/year afterward, consistent with a reduction in surgical activity during the COVID period followed by a rebound above the pre-pandemic rate.

**Figure 1:**
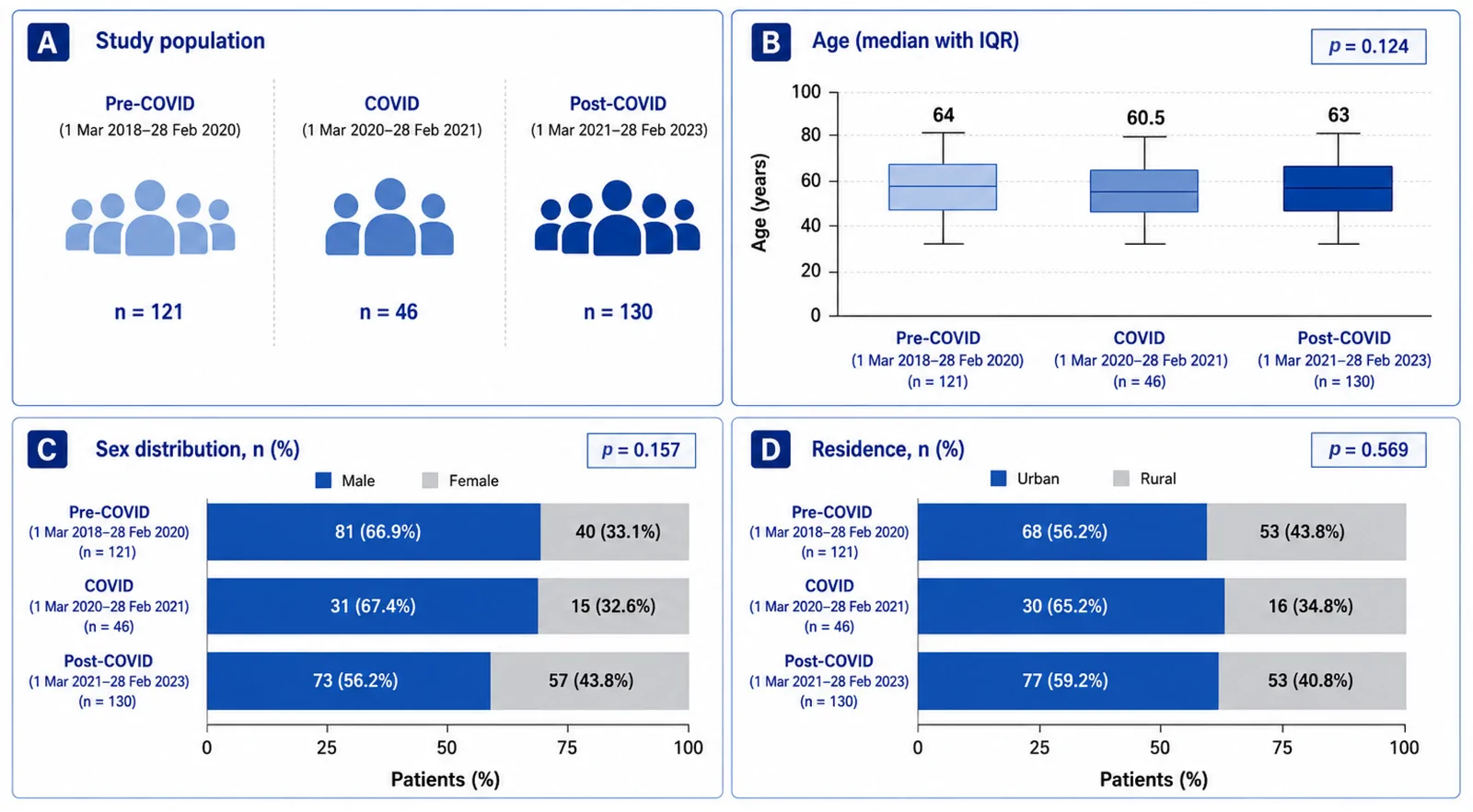
Patient demographics across study periods.

A male predominance was observed across all study periods, with males accounting for 66.9% of cases in the pre-COVID group (81/121), 67.4% during the COVID period (31/46), and 56.2% in the post-COVID group (73/130); the sex distribution did not differ significantly between groups (*p* = 0.157). The median patient age was similar across the three study periods: 64 years (IQR 54–71) in the pre-COVID group, 60.5 years (IQR 52–65) during the COVID period, and 63 years (IQR 56–68) in the post-COVID group (*p* = 0.129). Similarly, the distribution of place of residence was comparable between groups, with urban residents accounting for 56.2% of cases in the pre-COVID group (68/121), 65.2% during the COVID period (30/46), and 59.2% in the post-COVID group (77/130) (*p* = 0.569). Patient demographics are summarized in Figure 1.

Tumor size did not differ significantly across the three study periods, with median values of 5.5 cm in the pre-COVID group (IQR 3.5–7.6), 4.9 cm during the COVID period (IQR 2.9–6.9), and 5.5 cm in the post-COVID group (IQR 3.7–7.3) (*p* = 0.233). Regarding histological subtype, the vast majority of tumors were clear-cell RCC (258/297, 86.9%), while non-clear-cell subtypes were infrequent and included papillary RCC (12/297, 4.0%), chromophobe RCC (10/297, 3.4%), RCC not otherwise specified (NOS) (11/297, 3.7%), acquired cystic disease-associated RCC (3/297, 1.0%), MiT translocation RCC (2/297, 0.7%), and mucinous tubular and spindle cell carcinoma (1/297, 0.3%).

Pathological characteristics are summarized in Table 1. The proportion of high-stage tumors (pT3–pT4) differed significantly, increasing from 41.3% in the pre-COVID group and 34.8% during the COVID period to 61.5% in the post-COVID group (*p* < 0.001). Similarly, vascular invasion was significantly more frequent after the pandemic, being identified in 29.8% of pre-COVID cases, 34.8% during the COVID period, and 56.9% in the post-COVID group (*p* < 0.001).

To characterize the nature of this increase in more detail, vascular invasion was further subtyped into renal sinus vascular invasion, segmental/renal vein invasion, microscopic vascular invasion, and unspecified vascular invasion (Table 2); these categories were not mutually exclusive, as individual tumors could exhibit more than one type. The overall increase in vascular invasion was driven predominantly by segmental/renal vein invasion, which more than doubled between the pre-COVID and post-COVID periods (17.4% to 37.7%), while renal sinus invasion and microscopic vascular invasion increased only modestly. Notably, the proportion of unspecified vascular invasion cases declined over the study period (5.8%, 0%, and 0.8%, respectively), suggesting more systematic subtyping of vascular invasion in pathology reports over time; however, this category accounted for only a small minority of cases throughout, and its reduction is unlikely to fully account for the much larger increase observed specifically in segmental/renal vein invasion.

**Figure 2:**
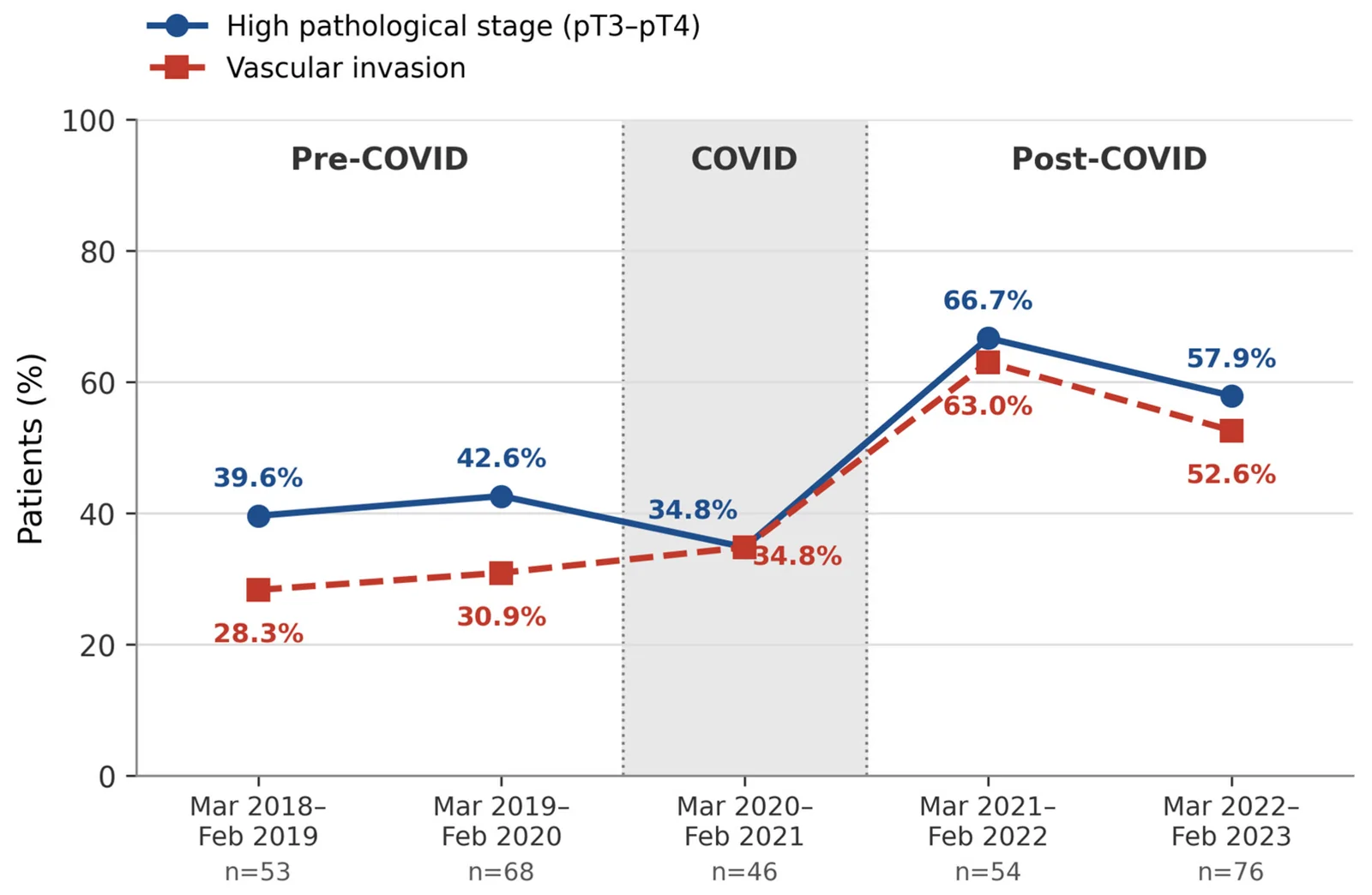
Annual trend in high pathological stage (pT3–pT4) and vascular invasion.

To evaluate whether the observed post-COVID shift reflected a discrete, pandemic-associated change rather than a pre-existing secular trend, pathological stage and vascular invasion were additionally examined on a year-by-year basis across the full study period (Table 3, Figure 2). The proportion of high-stage tumors and vascular invasion remained stable across the two pre-pandemic years (39.6% and 42.6% high-stage; 28.3% and 30.9% vascular invasion), decreased slightly during the pandemic year (34.8% for both), and then increased sharply in the first post-pandemic year (66.7% high-stage; 63.0% vascular invasion), with partial regression toward baseline in the second post-pandemic year (57.9%; 52.6%).

To further characterize the magnitude and precision of these associations, odds ratios (ORs) with 95% confidence intervals (CIs) were calculated for high-pT stage (pT3–pT4) and vascular invasion in the post-COVID group relative to the pre-COVID and COVID groups (Table 4). Post-COVID tumors were significantly more likely to present with high-pT stage compared with the pre-COVID group (OR 2.27, 95% CI 1.37–3.77) and the COVID group (OR 3.00, 95% CI 1.49–6.05). Similarly, vascular invasion was significantly more frequent in the post-COVID group compared with the pre-COVID group (OR 3.12, 95% CI 1.85–5.26) and, at the level of the unadjusted comparison, the COVID group (OR 2.48, 95% CI 1.23–4.99). Three of the four comparisons—both pT-stage comparisons and vascular invasion post-COVID vs. pre-COVID—remained statistically significant after Holm-Bonferroni correction for multiple comparisons across the ten pairwise tests performed (adjusted *p* ranging from <0.001 to 0.017). The comparison of vascular invasion between the post-COVID and COVID groups did not survive this correction (adjusted *p* = 0.077), indicating that this specific pairwise difference should be interpreted with caution given the smaller COVID-period sample size (*n* = 46) and the number of comparisons tested. In sensitivity analyses restricting the post-COVID period to individual study years, both high pathological stage and vascular invasion remained significantly more frequent than in the pre-COVID group under both alternative definitions tested, with associations that remained statistically significant after Holm-Bonferroni correction applied within this family of comparisons; exact case counts, odds ratios, 95% confidence intervals, and raw and adjusted *p*-values are reported in Supplementary Table S1. These results indicate that the findings were not dependent on the specific period boundary chosen.

To assess whether the association between the post-COVID period and these two outcomes was independent of other variables, multivariable logistic regression models were constructed with study period as the principal predictor, adjusted for age, sex, tumor size, residence, and histological subtype (Table 5). The post-COVID period remained independently associated with both high pathological stage (adjusted OR 2.91, 95% CI 1.61–5.25, *p* < 0.001) and vascular invasion (adjusted OR 3.80, 95% CI 2.11–6.83, *p* < 0.001) relative to the pre-COVID group, with both associations numerically stronger than in the corresponding unadjusted analyses. The COVID period was not significantly associated with either outcome relative to pre-COVID. Tumor size was independently associated with both outcomes (both *p* < 0.001), and non-clear-cell histological subtype was independently associated with lower odds of both high stage and vascular invasion (*p* = 0.008 and *p* = 0.019, respectively); age, sex, and residence were not significant predictors in either model. Both models were estimated on the full cohort (*n* = 297) without missing data among the included predictors, and demonstrated adequate overall fit (high-pT stage: χ^2^(7) = 91.4, *p* < 0.001, McFadden R^2^ = 0.222; vascular invasion: χ^2^(7) = 74.3, *p* < 0.001, McFadden R^2^ = 0.182).

In contrast, the prevalence of high WHO/ISUP grade (assessable in 284 of 297 cases) did not differ significantly among the study periods, occurring in 59.5%, 48.9%, and 60.2% of graded cases, respectively (*p* = 0.392). Likewise, tumor necrosis (35.5%, 32.6%, and 32.3%, respectively; *p* = 0.852) and sarcomatoid and/or rhabdoid differentiation (19.0%, 10.9%, and 18.5%, respectively; *p* = 0.433) showed no significant differences across the study periods. Consistent with these findings, odds ratios for these three variables were non-significant in all pairwise comparisons, with wide 95% confidence intervals crossing 1 (Table 4).

## 4. Discussion

The present study found that patients undergoing surgery for RCC in the post-COVID period more frequently presented with advanced pathological stage and vascular invasion compared with those treated before or during the pandemic, both established predictors of adverse oncological outcomes in RCC [12,13,14], though, as discussed below, these two findings are closely related rather than fully independent. In contrast, tumor size, WHO/ISUP grade, tumor necrosis, and sarcomatoid/rhabdoid differentiation, established markers of aggressive tumor biology associated with recurrence and cancer-specific mortality [12,15,16,17,18,19], and, in the case of necrosis, influenced by biological factors such as angiogenesis and intratumoral hypoxia [20], did not differ significantly across periods. This selective pattern, affecting features that reflect the anatomical extent of disease rather than markers more closely tied to intrinsic tumor biology, is compatible with a mechanism involving local tumor growth over a longer interval before treatment, rather than a general shift in tumor biology. However, given the retrospective, uncontrolled, single-center design of this study, these findings describe changes in the surgical cohort’s pathological profile over time and do not, on their own, establish that pandemic-related healthcare disruption caused them.

Published studies evaluating the pandemic’s impact on RCC have reported heterogeneous findings [6,7,8,9]: some identified a shift toward more advanced disease at presentation, while others found no significant change despite reductions in RCC diagnoses and surgical activity. This heterogeneity likely reflects differences in healthcare organization, referral pathways, and the duration and severity of restrictions across different systems [21,22,23]. Romania’s public healthcare system relies heavily on centralized, hospital-based referral for urological oncology, with comparatively limited outpatient imaging capacity outside major centers; more prolonged restriction of elective and diagnostic services nationally may partly explain why a measurable pathological shift emerged in our cohort despite its absence in the Italian and Dutch cohorts of Mangone et al. and Yildirim et al., where such services were reportedly restored more rapidly [8,9]. Differences in study design, patient selection, and the variables analyzed may also have contributed to this inconsistency [6,7,8,9]. Our study adds pathology-based data from an Eastern European tertiary referral center, a setting for which, to our knowledge, such data remain limited.

On an annual basis, the proportion of high-stage tumors and vascular invasion was stable across the two years preceding the pandemic, decreased slightly during the pandemic year, and then increased in the first year following the resumption of routine care, with partial regression in the subsequent year (Table 3, Figure 2). This pattern is not readily explained by a pre-existing secular trend, although changes in referral pathways, operative selection, pathology reporting practices, or institutional case mix over the same interval cannot be excluded, as discussed further below. The increase in stage was concentrated among tumors crossing the pT3a threshold, while pT1 tumors correspondingly declined and no escalation to pT3b, pT3c, or pT4 was observed; the concurrent increase in vascular invasion was driven predominantly by segmental/renal vein invasion. Because segmental/renal vein invasion is itself one of the histological criteria defining pT3a, these two findings are not fully independent: they substantially overlap, both reflecting the same anatomical feature—venous invasion—rather than representing separate confirmations of increased tumor aggressiveness. Both pathological stage and vascular invasion are nonetheless important, if overlapping, determinants of prognosis routinely considered in postoperative risk stratification for RCC [24,25], and vascular invasion specifically has been associated with increased risk of recurrence and poorer survival even after adjustment for other clinicopathological variables [14,26,27]. We cannot fully exclude a contribution from more systematic subtyping of vascular invasion in pathology reports over the study period, as the proportion of unspecified vascular invasion cases declined over time; however, the magnitude of the increase in segmental/renal vein invasion specifically is unlikely to be fully explained by this alone.

Several mechanisms could contribute to the observed shift. During the pandemic, healthcare resources were redirected toward managing infected patients, reducing outpatient consultations, diagnostic imaging, and elective surgical capacity; urological societies consequently recommended prioritizing surgery by oncological risk [28,29,30]. Because a substantial proportion of RCCs are detected incidentally on imaging performed for unrelated indications, reduced imaging utilization may also have delayed detection of otherwise asymptomatic tumors [31,32,33]. Institutional data on imaging volumes, outpatient referral rates, and diagnosis-to-surgery intervals were not available for this cohort, so this explanation remains inferential rather than demonstrated.

An alternative, non-mutually-exclusive explanation is a rebound or selection phenomenon: patients whose diagnosis or surgery was deferred during the pandemic may have been triaged toward earlier intervention according to perceived urgency once services resumed, over-representing advanced tumors among surgical cases during the catch-up period independent of any true increase in disease progression. We cannot exclude this possibility with the available data. However, total surgical volume in the final study year (76 cases) exceeded the pre-pandemic annualized rate (60.5 cases) at a time when the proportion of high-stage disease remained elevated (57.9%); a pure triage-driven selection process, in which lower-stage cases continued to be deferred, would be expected to coincide with persistently suppressed rather than normalized surgical volume. This observation offers some evidence against selection as the sole explanation, though it does not exclude a partial contribution, and prospective data on surgical waiting-list composition would be needed to confirm this.

More broadly, our annual descriptive analysis cannot exclude changes in referral pathways, operative selection criteria, pathology reporting practices, or institutional case mix over the study period, any of which could contribute to the observed pattern independent of a true increase in disease severity at the population level. Disentangling these possibilities from a delayed-diagnosis mechanism is not possible using a retrospective, single-center surgical-cohort design.

This study’s pathology-based, multi-period design allowed simultaneous evaluation of several established prognostic factors, but several limitations should be acknowledged. First, the retrospective design precludes causal inference and may be subject to selection bias and unmeasured referral-pattern effects. Second, this was a single-center study, which may limit generalizability. Third, to assess whether the association between study period and these outcomes was independent of other variables, multivariable logistic regression models were constructed for high pathological stage and vascular invasion, adjusting for age, sex, tumor size, residence, and histological subtype (dichotomized as clear-cell versus non-clear-cell RCC). The association between study period and both outcomes remained significant—and numerically stronger—after adjustment, indicating that these variables do not account for the observed findings. However, other potentially relevant variables, such as comorbidities, smoking status, or family history, were not captured in the pathology reports and could not be included, so residual confounding cannot be fully excluded; in addition, the small number of patients within individual non-clear-cell subtypes required these categories to be combined for the regression, which may obscure subtype-specific associations. Fourth, no systematic re-review of archival slides was performed across the 2016 and 2022 WHO classification editions spanned by this study; although grading and staging criteria were substantively stable between editions, subtle inter-observer or temporal variation in classification cannot be excluded. Fifth, institutional data on imaging volumes, outpatient consultations, referral pathways, and diagnosis-to-surgery intervals were not available, so the proposed mechanism linking pandemic-related healthcare disruption to delayed diagnosis remains inferential rather than directly demonstrated. Sixth, the post-COVID period as defined spans both a later-pandemic phase and a subsequent recovery phase rather than a single uniform interval; this heterogeneity is directly demonstrated by our year-by-year analysis and should be considered when interpreting findings attributed to the post-COVID period as a whole. Finally, long-term clinical follow-up data, including recurrence and cancer-specific survival, were not available; consequently, our findings describe the anatomical extent of disease at the time of surgery and should not be interpreted as demonstrating an effect on oncological outcomes.

By providing pathology-based evidence on RCC presentation across the COVID-19 pandemic from an Eastern European tertiary referral center, this study underscores the importance of maintaining timely diagnostic pathways and uninterrupted access to cancer care during future healthcare crises. Confirming whether the observed pathological differences translate into differences in recurrence and survival, and disentangling the alternative explanations discussed above, will require multicenter studies that combine long-term oncological follow-up with prospective data on referral and surgical scheduling.

## 5. Conclusions

This retrospective pathology-based study demonstrates a post-pandemic shift toward more advanced RCC at the time of surgical treatment, reflected by a significantly higher pathological stage and an increased prevalence of vascular invasion, a pattern that remained consistent across annual and sensitivity analyses. In contrast, WHO/ISUP grade, tumor size, tumor necrosis, and sarcomatoid/rhabdoid differentiation remained unchanged across the study periods. Collectively, these findings are temporally associated with the post-pandemic period and are consistent with, though do not establish, disruptions in healthcare delivery during the COVID-19 pandemic as a contributing factor to patients presenting with a greater anatomical extent of disease, without a corresponding change in the intrinsic histological aggressiveness of the tumors.

By providing pathology-based evidence from an Eastern European tertiary referral center, this study adds to the growing literature on the long-term oncological consequences of the COVID-19 pandemic. These findings underscore the importance of maintaining timely diagnostic pathways and uninterrupted access to cancer care during future healthcare crises. Further multicenter studies with long-term oncological follow-up are warranted to determine whether the observed pathological differences translate into differences in recurrence and survival.

## Figures and Tables

**Table 1:** Pathological characteristics by study period.

Variable	Pre-COVID (*n* = 121)	COVID (*n* = 46)	Post-COVID (*n* = 130)	*p*-Value
Tumor size, cm, median (IQR)	5.5 (3.5–7.6)	4.9 (2.9–6.9)	5.5 (3.7–7.3)	0.233
High-pT stage (pT3–pT4)	50 (41.3%)	16 (34.8%)	80 (61.5%)	<0.001
* High WHO/ISUP grade (G3–G4)	69 (59.5%)	22 (48.9%)	74 (60.2%)	0.392
Vascular invasion	36 (29.8%)	16 (34.8%)	74 (56.9%)	<0.001
Tumor necrosis	43 (35.5%)	15 (32.6%)	42 (32.3%)	0.852
Sarcomatoid/rhabdoid differentiation	23 (19.0%)	5 (10.9%)	24 (18.5%)	0.433

* Note: WHO/ISUP grade was not assessable in 13 patients (10 with chromophobe RCC, which is not eligible for WHO/ISUP grading, and 3 with grade not specified in the original report); percentages for this row are calculated among graded cases only (*n* = 116, 45, and 123 for the pre-COVID, COVID, and post-COVID groups, respectively). All other rows use the full group *n* shown in the column headers.

**Table 2:** Distribution of vascular invasion subtypes across study periods.

Vascular Invasion Subtype	Pre-COVID (*n* = 121)	COVID (*n* = 46)	Post-COVID (*n* = 130)
Renal sinus vascular invasion	11 (9.1%)	3 (6.5%)	13 (10.0%)
Segmental/renal vein invasion	21 (17.4%)	9 (19.6%)	49 (37.7%)
Microscopic vascular invasion	7 (5.8%)	4 (8.7%)	15 (11.5%)
Unspecified vascular invasion	7 (5.8%)	0 (0%)	1 (0.8%)

Note: vascular invasion subtypes were not mutually exclusive; tumors exhibiting more than one type of vascular invasion were included in each applicable category. Percentages were calculated using the total number of tumors within each study period as the denominator.

**Table 3:** Annual distribution of pathological tumor stage and vascular invasion.

Pathological Feature	Mar 2018–Feb 2019 (*n* = 53) *n* (%)	Mar 2019–Feb 2020 (*n* = 68) *n* (%)	Mar 2020–Feb 2021 (*n* = 46) *n* (%)	Mar 2021–Feb 2022 (*n* = 54) *n* (%)	Mar 2022–Feb 2023 (*n* = 76) *n* (%)
pT stage					
pT1a	10 (18.9%)	19 (27.9%)	18 (39.1%)	10 (18.5%)	18 (23.7%)
pT1b	11 (20.8%)	14 (20.6%)	7 (15.2%)	7 (13.0%)	9 (11.8%)
pT2a	9 (17.0%)	4 (5.9%)	5 (10.9%)	0 (0%)	5 (6.6%)
pT2b	2 (3.8%)	2 (2.9%)	0 (0%)	1 (1.9%)	0 (0%)
pT3a	21 (39.6%)	28 (41.2%)	16 (34.8%)	36 (66.7%)	43 (56.6%)
pT3b	0 (0%)	0 (0%)	0 (0%)	0 (0%)	0 (0%)
pT3c	0 (0%)	0 (0%)	0 (0%)	0 (0%)	0 (0%)
pT4	0 (0%)	1 (1.5%)	0 (0%)	0 (0%)	1 (1.3%)
Vascular invasion					
Present	15 (28.3%)	21 (30.9%)	16 (34.8%)	34 (63.0%)	40 (52.6%)
Absent	38 (71.7%)	47 (69.1%)	30 (65.2%)	20 (37.0%)	36 (47.4%)

**Table 4:** Odds ratios and 95% confidence intervals for pathological variables, post-COVID group vs. pre-COVID and COVID groups, with Holm-Bonferroni-corrected *p*-values.

Variable	Comparison	OR (95% CI)	Raw *p*	Adjusted *p* *
High-pT stage (pT3–pT4)	Post vs. Pre-COVID	2.27 (1.37–3.77)	0.0015	0.013
	Post vs. COVID	3.00 (1.49–6.05)	0.0022	0.017
Vascular invasion	Post vs. Pre-COVID	3.12 (1.85–5.26)	<0.001	<0.001
	Post vs. COVID	2.48 (1.23–4.99)	0.011	0.077 ^†^
High WHO/ISUP grade (G3–G4)	Post vs. Pre-COVID	1.03 (0.61–1.73)	0.91	1.00
	Post vs. COVID	1.58 (0.79–3.14)	0.19	1.00
Tumor necrosis	Post vs. Pre-COVID	0.87 (0.51–1.46)	0.59	1.00
	Post vs. COVID	0.99 (0.48–2.02)	0.97	1.00
Sarcomatoid/rhabdoid differentiation	Post vs. Pre-COVID	0.97 (0.51–1.82)	0.91	1.00
	Post vs. COVID	1.86 (0.66–5.19)	0.24	1.00

* Holm-Bonferroni correction applied across all 10 pairwise comparisons. ORs calculated using the normal approximation to the log-odds ratio. ^†^ Following the corrected vascular invasion counts (Table 1), this comparison no longer reaches significance after Holm-Bonferroni adjustment; all other significant associations are unaffected.

**Table 5:** Multivariable logistic regression analyses of high pathological stage and vascular invasion.

Predictor	High-pT Stage (pT3–pT4)		Vascular Invasion	
	Adjusted OR (95% CI)	*p*	Adjusted OR (95% CI)	*p*
Period: COVID vs. Pre-COVID	0.99 (0.44–2.26)	0.989	1.59 (0.72–3.55)	0.254
Period: Post-COVID vs. Pre-COVID	2.91 (1.61–5.25)	<0.001	3.80 (2.11–6.83)	<0.001
Sex: Female vs. Male	1.03 (0.59–1.79)	0.920	1.34 (0.78–2.31)	0.289
Residence: Rural vs. Urban	1.36 (0.79–2.34)	0.270	1.07 (0.63–1.83)	0.801
Histological subtype: non-ccRCC vs. ccRCC	0.32 (0.14–0.74)	0.008	0.38 (0.17–0.85)	0.019
Age (per 1-year increase)	1.02 (0.99–1.05)	0.156	1.01 (0.98–1.04)	0.417
Tumor size (per 1-cm increase)	1.54 (1.36–1.74)	<0.001	1.40 (1.26–1.56)	<0.001

Reference categories: Period = pre-COVID; Sex = Male; Residence = Urban; Histological subtype = ccRCC. Model fit—High-pT stage: χ^2^(7) = 91.4, *p* < 0.001; AIC = 336; McFadden R^2^ = 0.222. Vascular invasion: χ^2^(7) = 74.3, *p* < 0.001; AIC = 349; McFadden R^2^ = 0.182. Both models estimated on *n* = 297 patients with no missing data across the included predictors.

## Data Availability

The raw data supporting the conclusions of this article will be made available by the authors on request.

## Supplementary Materials

The following supporting information can be downloaded at: https://www.mdpi.com/article/10.3390/cancers18172812/s1, Table S1: Sensitivity analyses using alternative definitions of the post-COVID period, relative to the pre-COVID group (*n* = 121).

## Abbreviations

The following abbreviations are used in this manuscript:

COVID-19	Coronavirus disease 2019
RCC	Renal cell carcinoma
WHO	World Health Organization
ISUP	International Society of Urological Pathology
SARS-CoV-2	Severe acute respiratory syndrome coronavirus 2
SCJUPBT	“Pius Brinzeu” Emergency County Hospital Timisoara
H&E	Hematoxylin and eosin
IQR	Interquartile range
NOS	Not otherwise specified
MiT	Microphthalmia-associated transcription factor
AJCC	American Joint Committee on Cancer
UICC	Union for International Cancer Control
TNM	Tumor, Node, Metastasis staging system
OR	Odds ratio
CI	Confidence interval
