# Supplementary material for: Renal Cell Carcinoma After the COVID-19 Pandemic: Evidence of More Advanced Pathological Presentation from a Romanian Tertiary Center
Source: Cancers (Basel). 2026 Aug 30;18(17):2812. doi: 10.3390/cancers18172812 (PMC13565521; doi:10.3390/cancers18172812)
Supplement: Supplementary file 1 [file cancers-18-02812-s001.zip › cancers-4498832-supplementary.pdf]

Table S1. Sensitivity analyses using alternative definitions of the post-COVID period, relative to the pre-COVID group (*n* = 121).

Post-COVID Definition	Variable	Present/Absent (Sensitivity vs. Pre-COVID)	OR (95% CI)	Raw *p*	Adjusted *p* *
Sensitivity A: 1 March 2022-28 February 2023 only (*n* = 76)	High pT stage (pT3-pT4)	44/32 vs. 50/71	1.95 (1.09-3.49)	0.024	0.024
	Vascular invasion	40/36 vs. 36/85	2.62 (1.45-4.76)	0.0015	0.0045
Sensitivity B: 1 March 2021-28 February 2022 only (*n* = 54)	High pT stage (pT3-pT4)	36/18 vs. 50/71	2.84 (1.45-5.56)	0.0023	0.0046
	Vascular invasion	34/20 vs. 36/85	4.01 (2.04-7.89)	<0.001	0.0002

Counts are presented as [present]/[absent] for the sensitivity-definition group vs. the pre-COVID group (e.g., "44/32 vs. 50/71" indicates 44 present and 32 absent in the sensitivity group, and 50 present and 71 absent in the pre-COVID group). ORs calculated using the normal approximation to the log-odds ratio. *Holm-Bonferroni correction applied separately within this family of four comparisons (independent of the correction applied to the ten comparisons in Table 4).
